# Identification and Characterization of Two Novel *bla*_KLUC_ Resistance Genes through Large-Scale Resistance Plasmids Sequencing

**DOI:** 10.1371/journal.pone.0047197

**Published:** 2012-10-09

**Authors:** Teng Xu, Jun Ying, Xiaoding Yao, Yulong Song, Ping Ma, Bokan Bao, Weiyan Jiang, Xinmei Wu, Huifen Tou, Peizhen Li, Ping Ren, Jingxian Fei, Lei Yang, Qi Liu, Zuyuan Xu, Tieli Zhou, Liyan Ni, Qiyu Bao

**Affiliations:** 1 Institute of Biomedical Informatics/Zhejiang Provincial Key Laboratory of Medical Genetics, Wenzhou Medical College, Wenzhou, Zhejiang, People’s Republic of China; 2 The Key Laboratory of Mammalian Reproductive Biology and Biotechnology of the Ministry of Education, Inner Mongolia University, Hohhot, Inner Mongolia Autonomous Region, People’s Republic of China; 3 College of Biological Science, Agricultural University of China, Beijing, People’s Republic of China; Wadsworth Center, United States of America

## Abstract

Plasmids are important antibiotic resistance determinant carriers that can disseminate various drug resistance genes among species or genera. By using a high throughput sequencing approach, two groups of plasmids of *Escherichia coli* (named E1 and E2, each consisting of 160 clinical *E. coli* strains isolated from different periods of time) were sequenced and analyzed. A total of 20 million reads were obtained and mapped onto the known resistance gene sequences. As a result, a total of 9 classes, including 36 types of antibiotic resistant genes, were identified. Among these genes, 25 and 27 single nucleotide polymorphisms (SNPs) appeared, of which 9 and 12 SNPs are nonsynonymous substitutions in the E1 and E2 samples. It is interesting to find that a novel genotype of *bla*_KLUC_, whose close relatives, *bla*_KLUC-1_ and *bla*_KLUC-2_, have been previously reported as carried on the *Kluyvera cryocrescens* chromosome and *Enterobacter cloacae* plasmid, was identified. It shares 99% and 98% amino acid identities with Kluc-1 and Kluc-2, respectively. Further PCR screening of 608 *Enterobacteriaceae* family isolates yielded a second variant (named *bla*_KLUC-4_). It was interesting to find that Kluc-3 showed resistance to several cephalosporins including cefotaxime, whereas *bla*_KLUC-4_ did not show any resistance to the antibiotics tested. This may be due to a positively charged residue, Arg, replaced by a neutral residue, Leu, at position 167, which is located within an omega-loop. This work represents large-scale studies on resistance gene distribution, diversification and genetic variation in pooled multi-drug resistance plasmids, and provides insight into the use of high throughput sequencing technology for microbial resistance gene detection.

## Introduction

Multidrug resistant *E.coli,* a clinically significant pathogen, has become a major threat to human health all over the world[1]-[3]. It has become a major cause of hospital-acquired infections worldwide mostly due to rapid acquisition of resistance determinants by horizontal gene transfer via mobile genetic elements such as plasmids, integrons and transposons [4]. More and more chromosomally encoded antibiotic resistance genes emerge on mobile genetic elements, especially those carried on plasmids which are thus easily disseminated. Examples include the CTX-Ms family extend-spectrum β-lactamases (ESBLs), which are mainly produced by *Enterobacteriaceae* plasmids and have become extensively widespread enzymes in the past two decades[5]–[7]. Quinolone resistance in *Enterobacteriaceae* is commonly considered to be the result of chromosomal mutations [8]. However, recently, plasmid-mediated quinolone resistance (PMQR) has been discovered, which is related to a variety of genes, including *qep*A, *qnr*, *oqx*AB, *aac*(6')-*Ib-cr*; this resistance occurs through plasmid transfer among different *Enterobacteriaceae* strains [9], [10]. Many of these PMQR genes are accompanied by ESBLs and/or aminoglycoside resistance genes on the same plasmid [8]. The increase in number as well as the complicated composition and constitution of resistance genes on plasmids poses a potential threat for empirical treatment of infections. Although plasmids are the most prevalent carriers of antimicrobial resistance determinants, the total resistance genotypes and gene abundance on them are difficult to assess unless complete plasmid sequences are available. Compared with large scale detection of resistance gene profiles, PCR methods appear to be cumbersome and time consuming. Furthermore, they are unsuitable for detecting the relative abundance of resistance genes in mixed samples which could provide insight into resistance gene epidemic tendency.

The second-generation sequencing technologies are generally used to resequence genomes whose reference sequences are available [11], [12]. Solexa sequencing, as one of the second-generation sequencing techniques, has the property of high throughput and can be used to simultaneously sequence templates at a very large scale [13]. It has also been successfully used in *de novo* sequencing and the assembly of large eukaryotic and bacteria genomes[11], [14]–[16]. In this study, we applied high throughput parallel sequencing to investigate resistance gene distribution, diversification and genetic variation of plasmids of 320 strains of multidrug resistance *E. coli* (named E1 and E2, each consisting of 160 clinical *E. coli* strains isolated from different time periods). Comparative genomics analyses led us to identify all the genes associated with previously known antibiotic resistance, which in turn permitted investigation of new subtypes of resistance genes.

**Table 1:** The emerging resistance genotypes, redundancy and references in the two samples.

Antibiotic Class	Resistance Type	E1 mappedreads/E1 Depth	E2 mappedreads/E2 Depth	Reference Acc.(coordinate)^a^	Reference Length (nt)
β-lactams	*bla*PSE	74/5.88	0/0	FJ201777(3.932)	930
	*bla*CMY	493/31.37	279/17.73	AY743434(47.1195)	1149
	*bla*OXA	420/35.18	1488/124.47	GQ438248(414.1286)	873
	*bla*LAP	560/47.89	1/0.09	EU529984(1.855)	855
	*bla*KLUC	606/50.67	0/0	EF057432(1636.2508)	873
	*bla*CTX-M-1^b^	2140/178.96	3190/327.03	AM420303(2739.3611)	873
	*bla*CTX-M-9^c^	11619/971.62	7136/596.77	GQ385326(2116.2988)	873
	*bla*TEM	41081/3495.32	23511/2000.37	U37195(76.933)	858
Aminoglycosides	*aac*C4	356/32.11	271/24.43	FJ032247(764.1573)	810
	*aad*B	916/112.64	143/17.66	FJ855133(64.657)	594
	*aad*A1	1677/157.62	977/91.81	EF539213(85.861)	777
	*aad*A2	1401/129.70	806/74.62	GU001948(107.895)	789
	*aph*A1	2336/209.76	1228/110.34	FJ705806(33685.34497)	813
	*aac*A4	3238/375.27	4563/528.75	Y11948(188.817)	630
	*aac*C2	13720/1167.38	9690/824.51	FJ848785(8370.9227)	858
	*str*A	23891/2025.61	15673/1328.9	FN432031(37812.38672)	861
	*str*B	24848/2174.95	17144/1500.62	AF024602(3156.3989)	834
Macrolides	*mac*B	6472/243.82	4402/165.85	CP000836(88414.90351)	1938
	*erm*B	2486/246.96	2124/211.02	AJ972604(272.1006)	735
	*mph*K	8546/615.28	6811/490.38	GQ274931(1106.2119)	1014
Fluoroquinolones	*qnr*B	1/0.11	116/12.53	EF682134(5270.5947)	678
	*qnr*A	87/9.81	0/0	AB469045(5736.6389)	654
	*qep*A	377/27.90	191/14.15	FJ183463(8630.9616)	987
	*oqx*A	477/29.73	86/5.41	GQ497565(1077.2249)	1173
	*oqx*B	1574/36.49	252/5.84	GQ497565(2276.5425)	3150
	*qnr*S	997/110.82	324/36.11	GQ336885(1.657)	657
Sulfonamides	*sul*I	12240/967.04	9571/770.38	CU459141(3650620.3651543)	924
	*flo*P	14018/1255.65	9536/854.20	GQ463142(18510.19324)	815
Tetracyclines	*tet*M	972/37.04	0/0	FN550102(12123.14039)	1917
	*tet*A	4931/283.02	4940/283.56	FJ621588(29035.30306)	1272
	*tet*B	14893/869.07	7318/427.03	AP010961(96650.97900)	1251
Chloramphenicol	*flo*R	147/8.91	1535/92.46	AY517519(66.1277)	1212
	*cml*A	6275/350.23	5765/321.77	GQ388247(8845.10152)	1308
	*cat*	5633/625.94	2188/243.19	FJ389187(7574.8230)	657
Trimethoprim	*dhf*r1	253/39.29	37/5.79	FM179327(98.568)	471
Rifampin	*arr*-2	1098/148.55	1139/154.08	FN396877(5.525)	540

a the accession number and coordinate of the reference.

b,c Because the resistance genotypes obtained in his work are heterozygous which come from different bacterial strains and the members in the same CTX-Ms group share >97% identities with each other, the genotype represented here is the multi-allele mapping concensus genotype.

## Materials and Methods

### Bacterial Strains Collection, Plasmid Extraction and High Throughput Sequencing

A total of 928 clinical strains of the *Enterobacteriaceae* family, isolated from sputum, urine, pus or blood samples of patients, were collected in the First Affiliated Hospital of Wenzhou Medical College, spanning the years 2002 to 2010. Among these isolates, 320 strains are *E. coli* which isolated from the years 2002–2003 (160 strains) and the years 2008–2009 (160 strains). The other 608 strains included *K. pneumoniae* (113 strains), *S. Marcescens* (132 strains),*E. cloacae* (190 strains), *E. aerogenes* (99 strains) and *C. Freundii* (74 strains). The bacterial samples in this study were collected from an large anonymous database according to the protocols of Wenzhou Medical College Ethics Committee so that the detailed participants information could not be obtained. Initially, all the participants orally consented that the isolates can be anonymously used in scientific study. The strains were identified by the Vitek-60 microorganism auto-analysis system (BioMerieux Corporate, France). For the pooled plasmids sequencing, each clinical *E.coli* strain was overnight incubated independently in 5 ml Luria-Bertani broth at 37°C for about 16 hours to obtain the concentration of an optimum optical density (OD_600_ = 1.5±0.2). The cultures were pooled and 100 ml of mixed bacteria was used to extract the plasmids. Plasmids were extracted by alkaline lysis method described as previously [17]. According to the isolated periods, the bacteria were pooled as E1 (160 *E.coli* strains isolated from the years 2002–2003) and E2. (160 *E.coli* strains isolated from the years 2008–2009) and their plasmids were subsequently used for high-throughput sequencing by Illumina/Solexa technology, respectively.

**Table 2:** The SNPs of resistance genotypes emerging in the two samples.

Genotype	Ref Pos^a^	Ref Base^b^	E1^c^	E2^c^	AA Change^d^
*aad*A1	121	C	\	C/T	H = >Y
	126	T	\	T/C	No
	590	A	A/G	\	K = >R
	711	G	C/G	G/C	No
	738	T	\	T/C	No
*tem*	18	T	T/C	\	No
	396	T	T/G	\	No
*flo*P	159	T	T/C	T/C	No
	197	C	C/A	\	A = >E
	792	G	G/A	G/A	No
*ctx*-m-1 group	239	T	\	C/T	A = >V
	725	G	A/G	G/A	D = >G/G = >D
*aac*C4	684	C	\	C/T	No
*str*B	139	G	G/A	G/A	E = >K/E = >K
*aad*A2	8	T	A/T	A/T	E = >V/E = >V
	207	C	T/C	T/C	No
	598	G	G/A	\	G = >S
*aac*C2	223	C	C/T	C/T	No
	232	G	G/A	G/A	E = >K/E = >K
	251	T	\	T/C	E = >K
	270	A	A/G	A/G	No
	378	G	G/A	G/A	No
	429	A	A/G	A/G	No
	450	T	T/C	T/C	No
	528	C	C/G	C/G	No
	546	G	G/A	G/A	No
	795	T	T/C	T/C	No
	802	A	A/G	A/G	T = >A/T = >A
*qnr*S	494	C	\	C/T	S = >F
*ctx*-m-9 group	508	C	C/T	\	No
*aph*A1	230	A	\	A/C	E = >A
*aac*A4	337	T	T/A	T/C	E = >Q/W = >R
	434	G	G/A	\	R = >Q
	568	G	\	G/T	D = >Y

a The position of the reference gene.

b Nucleotide type of the reference gene at the corresponding position.

c SNPs emerging at corresponding samples and locus. The left nucleotide of the oblique line represents the most abundant base and the right one indicates the second most prevalent base. Backslash suggests that the locus has no SNP detected.

d Amino acid changes when the best nucleotide is substituted by the second best nucleotide at the corresponding locus. The E1 and E2 samples are separated by the oblique lines.

### Reference Resistant Gene Sequence Collection, Sequencing Read Mapping and SNP Detection

Antibiotic resistant protein sequences were collected from ARDB (Antibiotic Resistance Gene database, http://ardb.cbcb.umd.edu) [18]. CD-HIT (http://bioinformatics.ljcrf.edu/cd-hit) was used for clustering protein sequences [19]. TBLASTN was used to compare protein sequences with a nucleotide collection database of NCBI. Comparison of nucleotide sequences was made using BLASTN [20]. The collected gene sequences were assembled using Phred/Phrap/Consed software package [21]. Mapping sequencing reads onto references was performed using SOAPaligner 2.20 (2 mismatch per read permitted), and SOAPsnp1.03 was employed to detect and annotate SNPs [22], [23]. A SNP was identified when at least 3 same high quality bases different from other bases at the same locus were detected. The relative abundance (sequencing depth) for a certain gene was calculated through the cumulative nucleotide length of the mapped reads on the gene divided by the gene size. Other bioinformatics tools used in this study were written by Perl and BioPerl (http://www.perl.org/).

**Figure 1:**
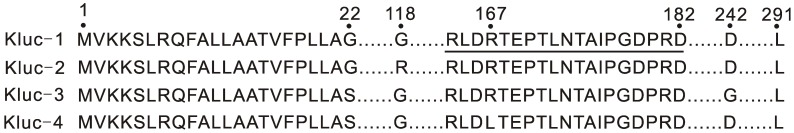
Amino acid sequence alignment of Kluc-3 and Kluc-4 with Kluc-1 from *K. cryocrescens* (GenBank accession No. AAK08976) and Kluc-2 from *E. Cloacae* (GenBank accession No. ABM73648). Dashes represent identical amino acid residues. The amino acids comprising the omega loop are underlined.

### *bla*_KLUC-3_ and *bla*_KLUC-4_ Positive Strain Screening, Cloning and Sequence Determination

The primers for positive strain screening and complete ORF cloning were designed according to the concensus sequence of *bla*_KLUC-3._ The screening primers were 5′-CGCTAAGCGTAGAGCAGAAACT-3′ and 5′- TCAGGTCACCCTTTTTGATCTC -3′ with a product of 228 bp in length. The primers for complete ORF cloning were 5′-GGGATCCATGGTTAAAA AATCATTACGCC -3′ and 5′-GAGATCTCTATAATCCCTCAGTGACGATT -3′ with a pair of flanking restriction endonuclease adapters (Bam HI for the forth primer and Bgl II for the reverse primer). Whole genomic DNAs of 928 clinical isolates of *Enterobacteriaceae* family were used as templates for PCRs. The complete ORF fragment of the PCR product was agarose-gel isolated and cloned into a pMD18 vector (TaKaRa). The recombinant clones were picked and sequenced by an ABI 3730 automated sequencer. The recombinant plasmid (pMD18::*bla*_KLUC_) was digested with Bam HI and Bgl II and the ORF fragment was recovered and further cloned into a pET28a vector (TaKaRa). Finally, the recombinant plasmids (pET28a::*bla*_KLUC_) were transformed into the host strain BL21. The complete ORF sequence of *bla*_KLUC-3_ and *bla*_KLUC-4_ have been deposited to GenBank with the accession numbers, JX185316 and JX185317, respectively.

**Table 3:** MICs of 18 β-lactams or antimicrobial drug complex for donors, transconjugants, transformants and the recipient control BL21.

Antibiotics	MIC(mg/L)						
	D41	Y214	TD41	TY214	BL21[pET28a :: kluc3]	BL21[pET28a :: kluc4]	BL21
Ampicillin	>512	>512	>512	>512	16	0.25	0.25
Aztreonam	64	128	64	128	0.03	0.03	0.03
Meropenem	0.03	0.03	0.03	0.03	0.03	0.03	0.03
Imipenem	0.5	0.25	0.5	0.25	0.5	0.5	0.5
Cefoxitin	16	64	4	64	1	1	1
Ceftriaxone	256	256	32	128	16	0.03	0.03
Piperacillin	128	512	32	512	1	0.25	0.25
Piperacillin + TZB	2	512	0.25	512	0.03	0.03	0.03
Cefazolin	>512	>512	>512	>512	64	1	1
Cefazolin + TZB	16	>512	1	>512	1	1	1
Cefuroxime	>512	>512	256	>512	32	0.5	0.5
Cefuroxime + TZB	64	>512	0.25	>512	0.25	0.25	0.25
Ceftazidime	32	512	32	512	0.06	0.06	0.06
Ceftazidime + TZB	0.5	256	0.03	256	0.03	0.03	0.03
Cefotaxime	256	128	32	128	8	0.03	0.03
Cefotaxime + TZB	0.25	32	0.03	32	0.03	0.03	0.03
Cefepime	64	64	8	64	0.5	0.03	0.03
Cefepime + TZB	0.25	16	0.25	16	0.03	0.03	0.03

### Conjugation and Susceptibility Test

Conjugation experiments were performed in mixed broth cultures. *E. coli* D41 and *E.cloacae* Y214 were used as the donors and rifampin-resistant EC600^Rifr^ was used as the recipient. Overnight cultures (incubated at 37°C with shaking) of the donor strain (500 µl) and the recipient strain (500 µl) were mixed together in 4 ml fresh Luria-Bertani broth and incubated for 6 hours at 37°C. The mixture was then inoculated onto a Trypticase soy agar (TSA) plate containing rifampin (Sigma; 512 mg/L) plus ceftriaxone (Roche; 2 mg/L) for 18 hours at 37°C. The colonies that grew on the selecting medium were picked and identified using the Vitek-60 system. The transferred plasmid was extracted and identified by PCR with *bla*_KLUC_ screening primers.

Minimal inhibitory concentrations (MICs) of 18 antibiotics or complex antimicrobial drugs were determined by the agar dilution method for two donors, two transconjugants, the recipient strain BL21, BL21[pET28a::*bla*_KLUC-3_] and BL21[pET28a::*bla*_KLUC-4_] in accordance with the guidelines of the Clinical and Laboratory Standards Institute (CLSI). Antimicrobial agents were obtained from the National Institute for the Control of Pharmaceutical and Biological Products (NICPBP) and pharmaceutical companies in China. *E. coli* ATCC 25922 was used as a quality control for the MIC determinations.

## Results

### Antibiotic Resistance Gene Distribution

To obtain relatively comprehensive reference sequences for the resistance genes, we collected the protein sequences from ARDB and our laboratory to complete a TBLASTN search against the nucleotide collection database (nt database) at NCBI with a constant E-value (1e-80). The internal fragments of nucleotide sequences which matched the proteins were then extracted. They were complete or partial Open Reading Frames (ORFs) of antibiotic resistance genes. To eliminate redundant members of this data set, we assembled these sequences and obtained 492 contigs (Table S1). They were used as “bait” references.

The samples E1 and E2, sequenced by Illumina Genome Analyzer, generated 819 and 694 million nucleotides, respectively. All the reads ranged from 73 to 75 nucleotides in length. Mapping reads onto the references yielded resistance genes and the quantity of mapped reads on a specific reference could also suggest the relative abundance of them in the sequenced samples. It showed that these two samples contained a total of 36 hits related to the resistance genes of β-lactams, aminoglycosides, macrolides, fluoroquinolones, sulfonamides, tetracyclines, chloramphenicol and rifampin. The most abundant gene was *blaTEM* with a sequence depth of 5496 folds (E1+E2) and the average sequence depth was 727 folds (Table 1). According to the ratio of mapped reads to the references, we found that resistance genes related to β-lactams and aminoglycosides were the most prevalent, not only in their total abundance, but also in their category of the corresponding genotypes. Of the 36 identified resistance genotypes, *bla*_PSE_, *bla*_KLUC_, *qnr*A and *tet*M were found only in the sample E1, while the other 32 types of resistance genes consistently appeared in both samples. The genotypes *bla*_TEM_, *str*B, *str*A, *flo*P, *aac*C2 and *sul*I were the most abundant genes in two samples.

### Polymorphism of the Resistance Genes

Polymorphism analyses revealed that 25 and 27 SNPs distributed in 9 and 10 resistance genes in E1 and E2 plasmid samples, respectively (Table 2). Among these 9 and 10 resistance genes, more than one half (5 and 7 genes, respectively) are associated with aminoglycosides resistance. Of the remaining 4 and 3 genes, 3 and 1 are involved in β-lactams resistance. Surprisingly, the genotype *aac*C2 (also known as *aac*(3)-*IIa*) in the E1 and E2 samples was scattered with 10 and 11 SNPs, respectively, whereas the *sulI* gene had an abundance approximately identical to *aac*C2 in the corresponding samples and did not show any SNP. Nine SNPs (36.0%) in E1 and twelve (44.4%) in E2 are nonsynonymous substitutions (Table 2). To demonstrate whether the sequencing reads were sufficient to reflect the SNP profiles, we used different fraction of total reads to identify SNPs. With the increased number of sequence reads mapped onto the references, the SNPs reached a stable stage (maximal number of SNPs corresponded to each sequencing library) when 60% of the total reads were used. It suggested that the sequencing data was sufficiently in depth to reflect a majority of the genetic variations from the two pooled plasmid samples.

### Identification of Two Novel *bla*_KLUC_ Genes

Kluc-1 has been identified as a close relative of CTX-M type class A ESBLs, sharing ∼85% amino acid identity with CTX-M-1 group members. It was first found in *Kluyvera cryocrescens* chromosome DNA [24]. Its variant, Kluc-2, one amino acid different from Kluc-1 at position 118, was demonstrated as a plasmid-mediated ESBL hosted in *E.cloacae* 7506 [25]. Mapping results indicated that some reads in the E1 sample matched to *bla*_KLUC_ reference with an average depth of 50.7. Further analyses showed that the potential *bla*_KLUC_ had 2 and 3 amino acids different from (sharing 99% and 98% amino acid identities with) Kluc-1 (AAK08976) and Kluc-2 (ABM73648), respectively. PCRs were performed against all 160 strains of the E1 sample to determine which strains carried this determinant. It showed that only strain *E. coli* D41 had a positive result. Full length ORF of the new *bla*_KLUC_ gene (named *bla*_KLUC-3_, GenBank Accession No. JX185316) was cloned into pMD18 vector and sequenced. The sequencing result is entirely identical to the mapping result of Solexa reads.

CTX-Ms, as one of the most prevalent β-lactamases with 90 known members, has been found in various bacteria of the *Enterobacteriaceae* family including *E. coli*, *K. pneumoniae*, *E. cloacae*, *S. enterica*, *E. aerogenes*, *C. Freundii*, *S. Marcescens*, *P. mirabilis,* etc [26]. Compared with other genotypes, the Kluc, with only 2 variants deposited in databases, seemed to appear rarely. To investigate the prevalence of the *bla*_KLUC_ gene and whether it appeared in the other genera of *Enterobacteriaceae* family besides *E. coli*, we screened 608 strains of clinical isolates collected from 2002 to 2010 in Wenzhou, China, including *K. pneumoniae* (113 strians),*S. Marcescens* (132 strains),*E. cloacae* (190 strains), *E. aerogenes* (99 strains), *C. Freundii* (74 strains) by PCR. Only one *E. cloacae* strain, named Y214, gave a positive result. Sequencing analysis of the new Kluc ORF showed 2, 3 and 2 amino acids different from Kluc-1, Kluc-2 and Kluc-3, respectively (Figure 1). This new subtype was thus named *bla*_KLUC-4_ (GenBank Accession No. JX185317).

### *bla*_KLUC-3_ and *bla*_KLUC-4_ Harbored on Plasmids

Conjugative experiments indicated that *bla*_KLUC-3_ or *bla*_KLUC-4_ harboring plasmids can transfer from donor cells (*E.coli* D41 and *E.cloacae* Y214) to the recipient *E.coli* EC600. This was further confirmed by sequencing of PCR products against plasmids extracted from the transconjugants. Both donor cells contain several plasmids ranging from about ∼10 kb to ∼150 Kb and the transconjugants showed similar plasmid profiles. The transconjugant of *E. coli* D41 (TD41) contained approximately 3 plasmids and the transconjugant of *E. cloacae* Y214 (TY214) contained about 4 plasmids. Sequencing of the plasmids is still in progress to determine which plasmids carry these *bla*_KLUCs_.

### Resistance Activities of *bla*_KLUC-3_ and *bla*_KLUC-4_

To detect resistance activities of *bla*_KLUC-3_ and *bla*_KLUC-4_, complete ORFs of these two novel resistant genes were cloned into pET28a vector and transformed into *E.coli* BL21. The MICs of the donors, the transconjugants, the transformants and the recipient controls against a group of antimicrobial drugs were detected (Table 3). BL21[pET28a::*bla*_KLUC-3_] showed resistance to several extend-spectrum β-lactams, including ceftriaxone, cefazolin and cefotaxime, but not ceftazidime. However, it is very interesting to find that the transformant BL21[pET28a::*bla*_KLUC-4_] did not show any resistance to the antimicrobial drugs examined. This may be largely attributed to the amino acid change at position 167, a substitution of Arg by Leu (R167L) in Kluc-4, leading to disappearance of the resistance activity (Figure 1). The β-lactamases inhibitor, for example tazobactam, could strongly reduce the activity of Kluc-3. This was consistent with previously described resistant activity of Kluc-1 [24]. The original *E. coli* D41 and *E. cloacae* Y214 have stronger resistance to and wider resistant spectrum of the antibiotics examined. Besides β-lactams, they are also resistant to tetracyclines, kanamycin and chloramphenicol.

## Discussion

In this work, we conducted a novel approach to detect resistance gene profiles in mixed *E.coli* plasmids isolated from two different periods of time. In order to effectively illustrate the composition of the resistome of the bacterial populations, 492 contigs as the universal “bait” references have been established. Because of the large number of resistance genotypes, as well as their subtypes or variants, in particular those with high identities, it would lead to formation of gene chimeras during the assembly process of the bait references and subsequently interfere with the effective estimation on genotypes and their SNP distributions. Therefore, once the reads mapped on bait reference, it should be manually checked and parsed, and the explicit genotype should be used for calculating resistance gene abundance and assigning SNPs, as listed in Table 1 column 5. On the other hand, the reads generated by Solexa sequencing is shorter in their sizes and the complete sequence of a certain resistance gene could not be entirely covered by single reads. Thus, the genotypes which harbored SNPs might be considered as heterozygous genotypes in the two pooled plasmid samples. The interested genes, such as *bla*_KLUC-3_, and their sequence characters would be screened and determined by PCR combined with Sanger sequencing.

Due to a strong ability to hydrolyze cefotaxime and high amino acid sequence similarities with CTX-M family enzymes, *bla*_KLUC_ was classified as one group of CTX-Ms family. Other groups of this family include CTX-M-1, CTX-M-9, CTX-M-8, CTX-M-25 and CTX-M-2, and the members in the same group share >94% identity, whereas ≤90% identity is observed between the members belongs to distinct groups [26]. Compared with many other class A ESBLs, such as those of TEMs, the CTX-Ms showed lower hydrolytic activities against penicillins and narrower anti-drug spectrum against cephalosporins. Its much lower hydrolytic activity against ceftazidime clearly distinguishes it from most enzymes in TEM and SHV families [26]. From our resistance susceptible test, we observed that cloned *bla*_KLUC-3_ showed resistance to several extended-spectrum cephalosporins, such as cefazolin, ceftriaxone, cefuroxime and cefotaxime, but not cefepime, ceftazidime, meropenem and aztreonam. Unlike *bla*_KLUC-3_, *bla*_KLUC-4_ under the same conditions as *bla*_KLUC-3_ did not show any resistance to the corresponding extended-spectrum cephalosporins. The MIC values for the examined β-lactams were the same as those of the recipient BL21 (Table 3). The obvious susceptibility difference to the β-lactams between two novel variants is probably related to a positively charged Arg residue replaced by a neutral residue Leu at position 167 which is an omega-loop (Ω-loop) comprising locus. The Ω-loop is a nonregular secondary structure most frequently occurring at the surface of a globular protein. It is involved in many protein functions, such as ligand, substrate or inhibitor-binding, tyrosine sulfation, as well as prohormonal cleavage[27]–[29]. More recently, a similar phenomenon has been observed from CTX-M-93 where only a single amino acid divergence from CTX-M-27 at position 169 (L169Q) located at an Ω-loop caused significantly decreased hydrolytic ability against its best substrates, such as cefotaxime, but led to enhanced resistance to ceftazidime [30].

It has been proposed that the *bla*_KLUC_ genes seem to be acquired from its chromosomal progenitor of the CTX-M-1 group [4]. The first discovered *bla*_KLUC-1_ was demonstrated encoded on chromosome, but the later identified variants, *bla*_KLUC-2,_ *bla*_KLUC-3_ and *bla*_KLUC-4_ were all harbored on the plasmids. This indicated that the movable DNA elements such as integrons, insert elements, transposons and plasmids might play essential roles in transposition and the horizontal gene transfer of the *bla*_KLUC_ resistance genes. It has been reported that ISEcp1 elements, the 42∼266 bp insertion sequences, could be involved in mobilization of CTX-M enzyme encoding genes [31], [32]. These well characterized and most widespread CTX-M enzymes include CTX-M-1, CTX-M-2, CTX-M-3, CTX-M-9, CTX-M-13, CTX-M-14, CTX-M-15, CTX-M-17, CTX-M-19, CTX-M-20 and CTX-M-2 [5], [33], [34]. To find whether the transposition of *bla*_KLUC-3_ and *bla*_KLUC-4_ is mediated in this manner, the upstream and downstream regions of this gene should be cloned and further analyzed.

To date, at least 90 types of β-lactams resistant genes and 1100 variants have been discovered [4]. The increased quantity of novel variants of each type sometimes creates different resistance phenotypes of decreased or increased susceptibilities to their substrates which have been demonstrated by their previously characterized relatives. In this work, we successfully found two novel *bla*_KLUC_ group members, and provided an example of a new resistance gene subtype screening and resistome investigation from pooled clinical isolates. Many mutagenesis-prone-to-happen sites of the gene locus were also detected and are mainly associated with β-lactams and aminoglycosides resistance. The merits of cost-effective and high-throughput of second-generation sequencing technology may have potential as a substitute, or a companion at least, for microarray in the field of large scale antimicrobial resistance gene detection.

## Supporting Information

Table S1 Available as supplementary data for this article.
(RAR)
